# Potential biomarkers relating pathological proteins, neuroinflammatory factors and free radicals in PD patients with cognitive impairment: a cross-sectional study

**DOI:** 10.1186/1471-2377-14-113

**Published:** 2014-05-22

**Authors:** Shu-yang Yu, Li-jun Zuo, Fang Wang, Ze-jie Chen, Yang Hu, Ya-jie Wang, Xiao-min Wang, Wei Zhang

**Affiliations:** 1 Department of Neurology, Beijing Tiantan Hospital, Capital Medical University, Beijing 100050, China; 2 Department of Geriatrics, Beijing Tiantan Hospital, Capital Medical University, Beijing 100050, China; 3 China National Clinical Research Center for Neurological Diseases, Beijing 100050, China; 4 Center of Parkinson’s Disease, Beijing Institute for Brain Disorders, Beijing 100053, China; 5 Beijing Key Laboratory on Parkinson’s Disease, Beijing 100053, China; 6 Core Laboratory for Clinical Medical Research, Beijing Tiantan Hospital, Capital Medical University, Beijing 100050, China; 7 Department of Clinical Laboratory Diagnosis, Beijing Tiantan Hospital, Capital Medical University, Beijing 100050, China; 8 Department of Physiology, Capital Medical University, Beijing 100069, China

**Keywords:** Parkinson’s disease, Cognitive impairment, Pathological proteins, Neuroinflammatory factors, Free radicals, Cerebrospinal fluid

## Abstract

**Background:**

Cognitive impairment strikingly reduces the quality of life of Parkinson’s disease (PD) patients. Studies find that pathological proteins, neuroinflammatory factors and free radicals may involve in the pathogenesis of cognitive impairment of PD, however, results are inconclusive.

**Methods:**

We recruited 62 PD patients and 31 healthy controls. PD patients were identified with cognitive impairment, including PD with mild cognitive impairment (PD-MCI) and PD with dementia (PDD) according to the diagnostic criteria for PD-MCI and PDD issued by Movement Disorder Society Task Force. The levels of pathological proteins, including β-amyloid 1–42 (Aβ1-42),Total-tau (T-tau) and phosphorelated tau (P-tau), neuroinflammatory factors,including tumor necrosis factor-α (TNF-α), interleukin-1β (IL-1β), interleukin-6 (IL-6), interferon-γ (INF-γ) and prostaglandin E_2_ (PGE_2_), free radicals, including hydroxyl radical (·OH), hydrogen peroxide (H_2_O_2_) and nitric oxide (NO) in cerebrospinal fluid(CSF) were detected. The levels of above factors in CSF were compared among healthy controls and patients with and without cognitive impairment. Correlation analyses were performed between Montreal Cognitive Assessment (MoCA) score and the levels of above factors in CSF.

**Results:**

T-tau level in CSF from PD-CI patients are significantly elevated comparing with those without cognitive impairment and controls (P = 0.016 and 0.004, respectively). The levels of P-tau (S396) and · OH in PD-CI patients are significantly higher than controls (P = 0.001 and 0.014, respectively). IL-6 levels in PD-CI patients are strikingly enhanced comparing with those without cognitive impairment (P = 0.005). MoCA score is negatively correlated with the levels of T-tau (r = -0.340), P-tau (S396) (r = -0.448), IL-6 (r = -0.489) and · OH (r = -0.504) in PD-CI patients.

**Conclusions:**

Elevated levels of T-tau, P-tau (S396), IL-6 and · OH in CSF are significantly correlated with cognitive impairment in PD patients. This investigation may suggest the potential biomarkers relating pathological proteins, neuroinflammatory factors and free radicals in PD patients with cognitive impairment.

## Background

Cognitive impairment (CI) is one of common non-motor symptoms in Parkinson’s disease (PD), which develops with the progression of PD. Mild cognitive impairment (MCI) is an early stage of CI in PD patients with an estimated prevalence of 20%-25% [1]. PD individuals with MCI have an increased risk of developing dementia, which occurs in the later stage of PD. The conversion rate from MCI to dementia in PD patients is about 6%-15% annually [2]. PD with dementia (PDD) strikingly reduces the quality of life of PD patients, enormously enhances financial burdens for their families and greatly causes life stresses for their caregivers. Early diagnosis and intervention of PD with MCI (PD-MCI) may delay its conversion to PDD.

Studies find that pathological hallmarks of Alzheimer’s disease (AD), such as β-amyloid (Aβ) and tau, are demonstrated to be linked with the risk of developing dementia [3] in PD patients. However, roles of above pathological proteins on PD-CI are not fully understood yet.

Neuroinflammation characterized by microglial activation serves as an engine driving PD progression. In substantia nigra, many endogenous and exogenous factors activate microglia and produce neuroinflammatory factors [4], such as tumor necrosis factor-α (TNF-α), interleukin-1β (IL-1β), interleukin-6 (IL-6), interferon-γ (INF-γ) and prostaglandin E_2_ (PGE_2_), which cause dopaminergic neuronal death. The dead neurons release iron, aggregated α-synuclein and neuromelanin into the extracellular spaces and provoke neuroinflammation by activating surrounding microglia [5], propagating progressive degeneration of dopaminergic neurons and deterioration of motor symptoms of PD. Recently, the significance of neuroinflammation in PD pathology extends beyond substantia nigra and neuroinflammation impairs regions relevant to non-motor symptoms [6]. However, there are few investigations on the role of neuroinflammation in the development and progression of PD with cognitive impairment (PD-CI) and the relationship between the pathological proteins and neuroinflammation in PD-CI is unclear yet.

Oxidative stress featured by the robust productions of highly toxic free radicals plays a pivotal role on cognitive decline in human with neurodegenerative diseases and PD animal model. An animal experiment shows positive correlations of spatial memory deficits with indicators of oxidative stress in rat PD model treated with lipopolysaccharide alone or plus 6-hydroxydopamine [7]. Lycopene protects against cognitive decline through inhibition of oxidative stress in rotenone-induced PD model [8]. These data indicate a critical role of oxidative stress on cognitive impairment in neurodegenerative diseases. However, there is no investigation on the relationships between PD-CI and free radicals in patients.

We hypothesize that the deposition of above pathological proteins in cognition-associated regions may, on one hand, activate microglia and produce neuroinflammatory factors, and on the other hand, cause oxidative stress and generate free radicals, leading to neuronal damage and cognitive impairment. To test this hypothesis, in this study, we assessed cognitive function for PD patients, detected the levels of pathological proteins, neuroinflammatory factors and free radicals in cerebrospinal fluid (CSF) from PD patients, and analyzed the relationships between cognitive impairment and above factors with aim to figure out the potential mechanisms and biomarkers associated with the development and severity of cognitive impairment in PD patients.

## Methods

### Subjects

We recruited 62 PD patients consecutively from the Department of Neurology, Beijing Tiantan Hospital, Capital Medical University from April 2010 to December 2013. Patients were diagnosed with PD according to UK Parkinson’s Disease Society Brain Bank criteria [9]. Total 36 patients had PD-CI, 33 of which were with MCI and 3 cases were with dementia according to the criteria for PD-MCI [10] and PDD [11], respectively. Cognitive functions of PD-CI patients were evaluated with the items from Montreal Cognitive Assessment (MoCA) and Mini-Mental Status Examination (MMSE), including sevens backwards, lexical fluency, clock drawing, pentagons and word recall, as well as informant interview and Pill Questionnaire. Patients with other primary explanations for cognitive impairment (e.g., delirium, stroke, major depression, metabolic abnormalities, adverse effects of medication, or head trauma) and PD-associated comorbid conditions (e.g., motor impairment or severe anxiety, depression, excessive daytime sleepiness, or psychosis) that, in the opinion of the clinician, significantly influence cognitive testing were excluded. The remaining 26 PD patients were without cognitive impairment (PD-NCI).

We recruited 31 normal controls consecutively based on the following criteria: no neurological symptoms and signs; no essential tremor, PD, secondary parkinsonism and Parkinson’s plus syndrome; no cognitive impairment and dementia; no systemic infectious diseases; no encephalitis, meningitis, cerebrovascular disease, brain tumors and other intracranial diseases; no surgical history; no dysarthria and mental illness that can effect expression; head MR is normal. The average age of control group was matched with that of PD group.

This study was approved by Beijing Tiantan Hospital Review Board. Written informed consents were obtained from participants or their guardians if they had dementia.

### CSF collection

Anti-parkinsonian drugs were withheld for 12–14 hours if patients’ condition allowed. We collected 3 ml CSF under fasting condition through lumbar puncture, followed by being centrifuged in 4°C at 3000 r/min for 10 minutes. Supernatant was then preserved at -80°C for detection.

### Detections of pathological proteins, neuroinflammatory factors and free radicals

#### Pathological proteins

The levels of Aβ1-42, T-tau and P-tau (S199, S396, T181 and T231) in CSF were detected by Enzyme-Linked Immuno Sorbent Assay (ELISA). CSB-E10684h kit for Aβ1-42 and CSB-E12011h kit for T-tau were from CUSABIO Company (China). KHB7041 kit for P-tau (S199), KHB7031 kit for P-tau (S396), KHO0631 kit for P-tau (T181) and KHB8051 kit for P-tau (T231) were from Invitrogen Company (USA).

#### Detection of neuroinflammatory factors

The levels of IL-6, IL-1β, TNF-α, INF-γ and PGE_2_ in CSF were detected by ELISA. 1R140 kit for IL-6, 1R040 kit for IL -1β, 1R350 kit for TNF-α and 1R330 kit for INF-γ were from RB Company (USA). CSB-E07965h kit for PGE_2_ was from CUSABIO Company (China).

#### Detection of free radicals

The levels of hydroxyl radical (·OH), hydrogen peroxide (H_2_O_2_) and nitric oxide (NO) in CSF were detected by chemical colorimetric method. A018 kit for · OH, A064 kit for H_2_O_2_ and A012 kit for NO were from Nanjing Jiancheng Biological Engineering Research Institute (China).

### Data analyses

Statistical analyses were performed with SPSS Statistics 20.0 (IBM Corporation, New York, USA). Demographic information and the levels of pathological proteins, neuroinflammatory factors and free radicals in CSF from the groups of control, PD-NCI and PD-CI were compared. Continuous variables, if they were normally distributed, were presented as means ± standard deviations and compared by ANOVA test. Bonferroni correction was performed in further comparisons between two groups, and the corrected P value was significant when it was <0.025. Continuous variables, if they were not normally distributed, were presented as median (quartile) and compared by nonparametric test. Discrete variables were compared by Chi square test. Spearman correlation analyses were made between MoCA score and the level of each pathological protein, neuroinflammatory factor and free radical in CSF from groups of PD-NCI and PD-CI. P value was significant when it was < 0.05.

## Results

### Clinical features of cognitive impairment in PD patients

In the total 62 PD patients, 36 (58.1%) cases are with PD-CI; 33 out of 36 PD-CI (53.2%) patients have MCI, and the remaining 3 patients (4.8% of total) have PDD. 26 (41.9%) PD patients are without cognitive impairment.

In PD-CI group, cognitive domain with the lowest scoring rate (actual score/total score) in MoCA is vocabulary memory (14.7%), followed by abstraction (39.7%), visuospatial and executive function (42.4%).

As shown in Table 1, there are significant differences in age and educational level among three groups. Further comparisons between two groups find that the average age of PD-CI group is significantly higher than that of control group (P = 0.006). Gender, onset lateralization, disease duration and H-Y stage have no significant difference among the three groups (P > 0.05).

**Table 1:** Demographic information and disease features of control, PD-NCI and PD-CI groups

**Demographic information and disease features**	**Control group (31 cases)**	**PD-NCI group (26 cases)**	**PD-CI group (36 cases)**	**P value**
Age (years, mean ± SD)	52.2 ± 8.6	57.4 ± 10.8	61.1 ± 9.4	0.019
Gender (male/total, %)	17/31 (54.8%)	13/26 (50.0%)	19/36 (52.8%)	0.483
Educational level				0.045
<6 years [case/total (%)]	11/31 (35.5%)	5/26 (19.2%)	19/36 (52.8%)	
6-12 years [case/total (%)]	15/31 (48.4%)	16/26 (61.5%)	12/36 (33.3%)	
>12 years [case/total (%)]	5/31 (16.1%)	5/26 (19.2%)	5/36 (13.9%)	
Onset lateralization				0.544
Left [case/total (%)]	-	11/26 (42.3%)	19/36 (52.8%)	
Right [case/total (%)]	-	13/26 (50.0%)	13/36 (36.1%)	
Both [case/total (%)]	-	2/26 (7.7%)	4/36 (11.1%)	
Disease duration [years, median (quartile)]	-	2.0 (2.0 ~ 4.5)	3.0 (1.0 ~ 5.0)	0.834
H-Y stage [stage, median (quartile)]		2.0 (1.0 ~ 2.5)	2.0 (1.5 ~ 2.6)	0.128

### The relationship between the levels of pathological proteins in CSF and cognitive impairment in PD patients

Table 2 shows the comparisons of the levels of T-tau and P-tau (S199, S396, T181 and T231) in CSF among groups of control, PD-NCI and PD-CI. Further correlation analysis indicates that MoCA score is negatively correlated with the levels of T-tau (r = -0.340,P = 0.009) and P-tau (S396) (r = -0.448, P = 0.036) in PD-CI patients. However, the levels of Aβ1-42 show no significant correlation with MoCA score.

**Table 2:** Comparisons of the levels of pathological proteins in CSF among control, PD-NCI and PD-CI groups

**Pathological proteins**	**Control group (31 cases)**	**PD-NCI group (26 cases)**	**PD-CI group (36 cases)**	**P**^**1**^	**P**^**2**^	**P**^**3**^
T-tau (pg/ml)	39.209 (10.274 ~ 141.454)	95.991 (23.105 ~ 125.394)	135.167 (41.261 ~ 175.683)	0.295	0.004	0.016
P-tau (S199) (pg/ml)	3.426 ± 0.561	6.544 ± 1.641	6.491 ± 1.579	0.000	0.000	1.000
P-tau (S396) (pg/ml)	49.335 ± 26.215	71.059 ± 22.399	76.454 ± 33.340	0.037	0.001	0.607
P-tau (T181) (pg/ml)	40.138 (30.722 ~ 53.644)	77.185 (49.271 ~ 95.730)	61.557 (55.461 ~ 84.649)	0.001	0.000	0.943
P-tau (T231) (pg/ml)	62.948 (46.241 ~ 80.793)	91.580 (87.163 ~ 148.387)	101.746 (71.852 ~ 180.582)	0.001	0.000	0.749
Aβ1-42 (ng/ml)	0.402 ± 0.082	0.432 ± 0.103	0.416 ± 0.113	0.273	0.590	0.535

P^**1**^: Control group vs. PD-NCI group; P^2^: Control group vs. PD-CI group, P^3^: PD-NCI group vs. PD-CI group.

### The relationship between the levels of neuroinflammatory factors in CSF and cognitive impairment in PD patients

Table 3 shows the comparisons of the levels of IL-6, IL-1β, TNF-α, INF-γ and PGE_2_ among the groups of control, PD-NCI and PD-CI. Correlation analysis indicates a significantly negative linear correlation between MoCA score and IL-6 level in CSF from PD-CI group (r = -0.489, P = 0.039). The levels of IL-1β, TNF-α, INF-γ and PGE_2_ are not dramatically correlated with MoCA score. Considering the influence of age on the levels of neuroinflammatory factors in CSF, we made further correlation analyses between IL-6 level in CSF and age in both PD group and PD-CI group, and find that the coefficients are not significant in both groups (r = 0.184, P = 0.401; r = 0.111, P = 0.731, respectively).

**Table 3:** Comparisons of the levels of neuroinflammatory factors in CSF among control, PD-NCI and PD-CI groups

**Neuroinflammatory factors**	**Control group (31 cases)**	**PD-NCI group (26 cases)**	**PD-CI group (36 cases)**	**P**^**1**^	**P**^**2**^	**P**^**3**^
IL-6 (pg/ml)	1.414 ± 0.295	1.382 ± 0.307	1.716 ± 0.187	0.084	0.025	0.014
IL-1β (pg/ml)	17.078 (14.578 ~ 18.943)	19.522 (16.759 ~ 31.843)	19.625 (17.792 ~ 29.010)	0.050	0.000	0.870
TNF-α (pg/ml)	139.227 (78.938 ~ 168.261)	77.356 (34.580 ~ 180.960)	71.480 (36.467 ~ 147.145)	0.328	0.006	0.392
INF-γ (pg/ml)	9.067 ± 2.496	6.695 ± 1.712	6.601 ± 2.049	0.027	0.003	0.935
PGE_2_ (pg/ml)	6.105 (5.564 ~ 8.960)	6.550 (5.589 ~ 13.771)	6.973 (4.977 ~ 16.392)	0.337	0.730	0.775

P^1^: Control group vs. PD-NCI group; P^2^: Control group vs. PD-CI group, P^3^: PD-NCI group vs. PD-CI group.

### The relationship between the levels of free radicals in CSF and cognitive impairment in PD patients

Table 4 shows the comparisons of the levels of ·OH, H_2_O_2_ and NO among the groups of control, PD-NCI and PD-CI. Correlation analysis indicates a significantly negative linear correlation between MoCA score and ·OH levels in CSF from PD-CI group (r = -0.504, P = 0.024). The levels of H_2_O_2_ and NO in CSF are not dramatically correlated with MoCA score in all groups.

**Table 4:** Comparisons of the levels of free radicals in CSF among control, PD-NCI and PD-CI groups

**Free radicals**	**Control group (31 cases)**	**PD-NCI group (26 cases)**	**PD-CI group (36 cases)**	**P**^**1**^	**P**^**2**^	**P**^**3**^
OH (U/ml)	666.518 ± 97.447	726.447 ± 87.393	745.943 ± 93.861	0.080	0.005	0.582
H_2_O_2_ (mmol/L)	1.973 (1.184 ~ 4.004)	1.973 (1.514 ~ 3.028)	2.154 (1.973 ~ 7.864)	0.797	0.265	0.479
NO (mmol/L)	49.767 (25.912 ~ 60.850)	69.905 (33.194 ~ 108.553)	68.481 (49.469 ~ 110.157)	0.024	0.000	0.530

P^1^: Control group vs. PD-NCI group; P^2^: Control group vs. PD-CI group, P^3^: PD-NCI group vs. PD-CI group.

### Correlations of the levels of neuroinflammatory factors and free radicals with pathological proteins in CSF from PD patients

No significant correlations are found between the levels of neuroinflammatory factors or free radicals and the levels of pathological proteins in CSF from PD patients.

## Discussions

In this study, 58.1% PD patients had cognitive impairment, which is roughly consistent with a result from another study (57% at a mean of 3.5 years from PD diagnosis) [12], demonstrating that cognitive impairment is a very common non-motor symptom in PD patients.

MoCA is a very sensitive scale for identifying the early cognition decline in PD-CI patients. It has high test-retest and inter-rater reliability and is notably correlated with a neuropsychological battery. In this study, the average scoring rate (actual score/total score) of MoCA in PD patients is 64.8%. Based on the scoring rate of all cognitive domains in MoCA from low to high level, dysfunction of vocabulary memory ranks the top in PD-CI patients, which is reported to be associated with hippocampus atrophy [13]. Abstraction is the second domain impaired significantly, followed by visuospatial and executive function. Disturbances of abstraction and executive function are associated with the impairment of frontal-striatal dopaminergic pathway [14]. Relationship between executive dysfunction and visuospatial deficit in PD patient is complicated [15]. Investigators have found that statistically controlling deficits in executive skills through analysis of covariance eliminates visuospatial impairment, implying that executive dysfunction may account for visuospatial impairment in PD subjects, however, statistically controlling visuospatial deficits fails to alter abnormal executive function, indicating that visuospatial dysfunction is independent of executive dysfunction in PD [16]. Additionally, posterior brain regions are found to be dominant for visuospatial ability [17].

Evidence shows that a short time to cognitive impairment is associated with old age [18], which is confirmed by our study. However, we find that educational level in PD-NCI group is higher than that in PD-CI group, which suggests that higher educational has a protective effect against cognitive impairment [19]. Gender, disease duration and H-Y stage are not the contributors to PD-CI in our study.

Increased T-tau level in CSF from patients with Alzheimer’s disease and other neurodegenerative diseases is widely thought to signify neuronal damage. However, few studies focus on T-tau level in CSF and PD-CI. One prospective study fails to find an association between T-tau level in CSF and cognitive decline in PD patients [20]. In this study, T-tau level in CSF in PD-CI group is significantly enhanced comparing with control group and PD-NCI group, and is increasingly elevated as cognition declines in PD-CI group, suggesting that T-tau may indicate the severity of PD-CI as a biomarker. P-tau is found in AD patients, however, there is no research on the relationship between P-tau level in CSF and PD-CI so far. Here, the levels of P-tau (S199), P-tau (T181) and P-tau (T231) in CSF in both PD-CI and PD-NCI groups are significantly increased comparing with control group, but there is no difference between PD-NCI group and PD-CI group. These data imply that P-tau (S199), P-tau (T181) and P-tau (T231) are more relevant to PD itself rather than cognitive impairment. However, P-tau (S396) level is significantly increased in PD-CI group comparing with control group, and moreover, MoCA score is negatively correlated with the level of P-tau (S396) in PD-CI group, suggesting that P-tau (S396) may be indicative of cognition deterioration in PD patients.

A prospective study has found that a lower baseline level of Aβ1–42 in CSF is associated with rapid cognition decline in PD patients [20]. Aβ1–42 level in CSF from PDD group is lower than non-demented PD and healthy control groups [21]. We do not find an association between PD-CI and Aβ1–42 level in CSF. Pathologically, PD-CI patients display the heterogeneity of amyloid-related changes [22], which may be reflected by the discrepancy in terms of the Aβ1–42 level in CSF. Data from our investigation may support the pathological heterogeneity of PD-CI in this group of patients, which is featured by the significantly elevated levels of T-tau and P-tau (S396) and the unchanged level of Aβ1–42.

Neuroinflammation featured by microglial activation contributes to the cascade events leading to neuronal degeneration in PD [23]. Varies of endogenous and exogenous factors, such as α-synuclein and lipopolysaccharide, activate microglia and produce a wealth of cytotoxic neuroinflammatory factors, including IL-6, IL-1β, TNF-α and INF-γ, etc. Neuroinflammatory factors exert toxic effects directly by binding to related receptors and activating second messenger pathways, or indirectly by inducing the expression of cyclooxygenase 2, a PGE_2-_generating enzyme [23], promoting degeneration and death of dopaminergic neurons and subsequent occurrence of motor symptoms.

Elevated levels of TNF-α [24], IL-1β [25] and IL-6 in CSF from PD patients have been reported [26]. However, role of neuroinflammation in the occurrence and development of PD-CI is rarely conducted. Aging is a risk factor for both PD and cognitive decline. Neuroinflammation becomes severer with age [27] and may underlie the cognitive disabilities in PD patients. Epidemiological data indicate a slightly inverse relationship between TNF-α level and cognitive function in the 50 to 60-year old of healthy subjects [28]. Chronic inflammatory disease exerts detrimental effects on cognitive function for persons with chronic periodontal inflammation [29]. High IL-1 level in hippocampus impairs learning ability [30]. Blocking IL-1 with receptor antagonist or knocking out IL-1 in mice [31] attenuates cognitive dysfunction. These data imply a potential correlation between neuroinflammation and cognitive impairment. However, role of neuroinflammation on PD-CI are rarely conducted. A PET scan with PK-11195, a ligand of peripheral binding site of benzodiazepine indicative of microglial activation, reveals neuroinflammation in PD patients [32]. Cognitive disorders-related cortical regions, including frontal and temporal lobes, have increased binding of PK-11195. Another study shows that PDD patients have significantly higher level of C-reactive protein in CSF than non-demented PD patients after controlling for age, gender and somatic illness [33]. In this study, IL-6 level in CSF in PD-CI group is not only prominently enhanced comparing with PD-NCI group, but also has a strikingly negative correlation with MoCA score, indicating that IL-6 may be a potential neuroinflammatory biomarker for the development and severity of cognitive impairment in PD patients. Considering the influence of age on the levels of neuroinflammatory factors in CSF, we made further analyses between IL-6 level in CSF and age in both PD group and PD-CI group, and find no significant correlation, which suggest that the elevated levels of neuroinflammatory factors are not resulted from aging, and are closely associated with PD-CI.

However, neuroinflammation is a complex network, in which each neuroinflammatory factor plays a different role through acting on different receptors and/or by targeting different signaling pathways at different stage. In this study, TNF-α and INF-γ may play compensatory roles on the deterioration of PD-CI since their levels in CSF in PD-CI group are decreased comparing with control group. However, the highly toxic effect of IL-6 may overweight the potential compensatory effect of TNF-α and INF-γ, leading to cognitive impairment eventually.

Oxidative stress characterized by robust generations of free radicals plays a crucial role on neuronal damage in PD. Upon a variety of stimuli, activation of microglial nicotinamide adenine dinucleotide phosphate oxidase 2, a superoxide-generating enzyme, produces a large amount of superoxide very quickly. Superoxide can be synthesized into H_2_O_2_, which induces the formation of fairly reactive and toxic •OH radical in the presence of a high concentration of free iron, causing damage to neurons. Microglial expression of inducible nitric oxide synthase leads to excessive NO generation [23]. Previous studies indicate an important role of oxidative stress on cognitive impairment in neurodegenerative diseases [7,8,34]; however, less attention is paid to the role of free radicals on PD-CI. In this investigation, •OH level in CSF in PD-CI group is significantly increased comparing with control group, and is negatively correlated with MoCA score, suggesting a pivotal effect of oxidative stress in PD-CI and •OH may be a potential indicator of PD-CI deterioration. Both PD-CI group and PD-NCI group have drastically higher NO levels in CSF than control group, implying that NO is relevant to PD regardless of the existence of cognitive impairment.

Correlations of the levels of neuroinflammatory factors and free radicals with pathological proteins in CSF in PD patients are not observed in this study. A variety of stimuli can induce neuroinflammation and oxidative stress in brain. Thus, pathological protein alone may not be sufficient to elicit robust productions of neuroinflammatory factors and free radicals in CSF.

## Conclusions

This study is the first part of our whole project and mainly focuses on PD-MCI, thus, few PDD patients were recruited within the limited time. We will enhance the sample size of PDD patients and investigate the clinical feature and potential mechanism of dementia in PD patients in the very near future.

In summary, PD patients have a high incidence of cognitive impairment, which mainly involves the cognitive domains of vocabulary memory, abstraction, visuospatial and executive function and language. The elevated levels of T-tau, P-tau (S396), IL-6 and ·OH in CSF are significantly correlated with PD-CI. This investigation may suggest the potential biomarkers relating pathological proteins, neuroinflammatory factors and free radicals in PD patients with cognitive impairment.

## Abbreviations

PD: Parkinson’s disease; CI: Cognitive impairment; MCI: Mild cognitive impairment; PD-NCI: Parkinson’s disease without cognitive impairment; PD-MCI: Parkinson’s disease with mild cognitive impairment; PDD: Parkinson’s disease with dementia; CSF: Cerebrospinal fluid; TNF-α: Necrosis factor-α; IL-1β: Interleukin-1β; IL-6: Interleukin-6; INF-γ: Interferon-γ; PGE2: Prostaglandin E_2_; MoCA: Montreal Cognitive Assessment; ELISA: Enzyme-Linked Immuno Sorbent Assay; OH: Hydroxyl radical; H2O2: Hydrogen peroxide; NO: Nitric oxide; AD: Alzheimer’s disease.

## Competing interests

The authors declare that they have no competing interests.

## Authors’ contributions

Research project: A. Conception: WZ, XW, YW; B. Organization: WZ; C. Execution: SY, LZ, FW, ZC and YH. Statistical Analysis: SY. Manuscript preparation: A. Writing of the first draft: SY; B. Review and Critique: WZ, LZ, FW, ZC and YH. All authors read and approved the final manuscript.

## Pre-publication history

The pre-publication history for this paper can be accessed here:

http://www.biomedcentral.com/1471-2377/14/113/prepub
